# Photovoltaic pumping tests: A novel supervision method for photovoltaic water pumping systems

**DOI:** 10.1016/j.heliyon.2024.e39718

**Published:** 2024-10-25

**Authors:** Ange Sahuquet, Simon Meunier, Judith A. Cherni, Anne Charpentier, Thomas Vezin, Arouna Darga, Guillaume Zuffinetti, Peter K. Kitanidis, Loïc Quéval

**Affiliations:** a Université Paris-Saclay, CentraleSupélec, CNRS, GeePs, 91192, Gif-Sur-Yvette, France; b Sorbonne Université, CNRS, GeePs, 75252, Paris, France; c Centre for Environmental Policy, Imperial College London, London, SW7 2AZ, United Kingdom; d Institut Photovoltaique d’Ile de France, UMR-IPVF 9006, CNRS, Ecole Polytechnique IPP, ENSCP PSL, Palaiseau, France; e DargaTech SARL, Ouagadougou, Burkina Faso; f Department of Civil and Environmental Engineering, Stanford University, Stanford, United States

**Keywords:** Photovoltaic systems, Energy-water nexus, Sustainability, Developing countries

## Abstract

Water pumps powered by photovoltaic energy, often named ‘photovoltaic water pumping systems’ (PVWPS), offer a promising solution for improving water access in developing regions. Regular pumping tests are essential for characterizing boreholes and ensuring sustainable groundwater extraction. Traditionally, these tests are conducted only at the time of PVWPS installation using diesel pumps. However, since PVWPS typically have a lifespan of around 20 years, the borehole's condition may change over time, necessitating ongoing testing. To overcome this challenge, this article presents a novel method for conducting pumping tests using the PVWPS's own photovoltaic modules as the power source, greatly simplifying regular borehole monitoring over the PVWPS's lifespan. This approach improves the long-term technical sustainability of PVWPS. By eliminating the need for diesel generators, it reduces also costs, emissions, and logistical complexity while ensuring continuous water supply during testing. The principle and protocol for these proposed tests are outlined, as well as the key indicators for analysis. Furthermore, the associated costs and benefits are thoroughly explored. The proposed method is applied to a PVWPS in a village in Burkina Faso. This PVWPS has 750 W_p_ of photovoltaic modules, a 10 m³ water tank, and a 56 m borehole. Results show that the photovoltaic pumping tests allow to accurately determine borehole parameters, achieving a model fit with an average R^2^ of 0.99. Additionally, a photovoltaic pumping test costs $43, which is significantly lower than standard pumping tests: a multiple step drawdown test costs $511 and a long pumping test costs $2050. Moreover, the proposed photovoltaic pumping tests can prevent premature replacements of PVWPS components, leading to significant savings. While demonstrated in a specific context, this method is transferable to other systems, offering potential benefits for companies, local authorities, governments, and NGOs involved in the development and maintenance of PVWPS in rural areas.

**Table:** Nomenclature

***Abbreviations***	
PV	Photovoltaic
PVWPS	Photovoltaic water pumping system
***Symbols***	
BDHI	Borehole dynamic health indicator
$C_{tot}$	Total cost of photovoltaic pumping test ($)
$C_{manpower}$	Manpower cost ($)
$C_{equipment}$	Equipment cost ($)
$C_{transport}$	Transport cost ($)
$C_{diesel}$	Average diesel cost for the considered country (€/L)
$Co_{diesel}$	Diesel consumption of the vehicle (L/km)
d	Distance to be travelled from the closest urban area (>100 000 inhabitants) to PVWPS location (km)
ΔT	Parameter representing the characteristic response time of the borehole (min)
$\eta_{mp}$	Motor-pump efficiency
$H_{b}\left(t\right)$	Borehole water depth (m)
$H_{b,d}\left(t\right)$	Borehole dynamic water depth (m)
$H_{b,d,sim\;}$	Borehole dynamic water depth simulated using photovoltaic pumping test parameters (m)
$H_{b,d,0,sim\;}$	Borehole dynamic water depth simulated using initial pumping test parameters (m)
$H_{b,s}$	Borehole static water depth (m)
$H_{b,s,0}$	Borehole static water depth determined during the initial pumping test (m)
$H_{b,sim}$	Simulated borehole water depth (m)
$H_{b,meas}$	Measured borehole water depth (m)
$H_{in,t}$	Height between the ground level and the level at which water enters the tank (m)
$\kappa_{n}$	*n*^th^ linear coefficient of the borehole model (m^−2^.s)
N	Fitting parameter representing the effect of the past flows on the borehole water depth
NRMSE	Normalized root mean square error
$\mu_{n}$	*n*^th^ quadratic coefficient of the borehole model (m^−5^.s^2^)
$P_{p}$	PV modules peak power in standard test conditions (W_p_)
$Q_{p}\left(t\right)$	Pumped flow rate (m^3^.s^−1^)
$\tilde{Q_{p}}$	Characteristic pumped flow rate (m^3^.s^−1^)

## 1 Introduction

Lack of water access in rural off-grid areas represents a severe challenge in developing countries [1,2]. Water pumping systems powered by photovoltaic energy are a promising solution to address the problem [3,4]. They are economically competitive in remote locations [5] and do not emit greenhouse gases during operation [6]. In sub-Saharan Africa, because irradiance is large and main electricity grid coverage is low [7,8], photovoltaic water pumping systems (PVWPS) which extract groundwater are good candidates to improve particularly domestic water access [9], and are promoted by numerous governments, non-governmental organizations (NGOs) and development institutions [10,11]. These systems were also identified as an interesting solution to improve access to irrigation in rural areas [12,13]. However, because they collect water directly from groundwater resources, they need to be monitored in order to avoid over-extraction and guarantee groundwater sustainability [14,15]. In addition, it is paramount to guarantee the longevity of the system, and a long-term access to water for its users [16,17].

Pumping tests are one of the main tools to ensure that water extraction from a pumping system is viable [16,18]. They consist in pumping water at a given flow rate for a certain duration and measuring the response of the water depth in the borehole [19]. They allow to derive information about the state of health of the borehole (e.g., clogging) and the maximum flow rate that can be extracted from the borehole, in order to ensure a sustainable sizing of the system [20,21]. In the frame of PVWPS for domestic water access and irrigation in developing regions, pumping test are usually performed only at the PVWPS installation (more specifically, just after the borehole drilling or the recommissioning of an existing borehole), using a diesel pump to extract water at various flow rates [22,23]. This raises a problem because the lifetime of a PVWPS is ∼20 years [24,25] and the borehole state of health may vary over this lifetime.

In order address this problem, a method is proposed in this article to regularly perform pumping tests during the lifetime of a PVWPS at a minimal cost. These tests use the photovoltaic (PV) panels of the PVWPS as the energy source to extract water instead of a diesel generator and are thus named “photovoltaic pumping tests” in the rest of the article. By opposition, the pumping tests that are usually done are referred as “standard pumping tests”.

In Section 2, a review of the literature is provided, along with an overview of the PVWPS architecture, risks associated with groundwater pumping, and the current use of pumping tests. The novel contribution of this article is also described. Section 3 introduces the concept and cost of the proposed photovoltaic pumping tests. In Section 4, the results of standard pumping tests and photovoltaic pumping tests are presented for a PVWPS installation in a village in Burkina Faso. This allows to quantify the costs and benefits of performing photovoltaic pumping tests in this case.

The objective of this work is to develop and assess the newly proposed approach for evaluating the sustainability of PVWPS boreholes over their lifespan: photovoltaic pumping tests. More specifically, the relevance of these new tests is explained, and the principles and protocols for conducting them are detailed. Additionally, the key indicators selected for analysing these tests are presented, along with a cost-benefit evaluation of their implementation.

## 2 Literature review

In Section 2.1, the literature on photovoltaic water pumping systems is reviewed, and in Section 2.2, the focus is on the literature concerning the interaction between pumping systems and groundwater resources, particularly with respect to the drawdown and the borehole behaviour during pumping. Section 2.3 then reviews the literature on standard pumping tests, such as multi-step drawdown test and long-term tests, typically conducted before photovoltaic water pumping systems (PVWPS) are installed, along with an analysis of the costs and challenges associated with these tests. In Section 2.4, the necessity of regular borehole monitoring and the relevant legislative frameworks are presented. Finally, in Section 2.5, the novel contribution of this article is outlined, highlighting how it enhances the monitoring of PVWPS boreholes.

### 2.1 Photovoltaic water pumping systems

In rural areas of developing countries, photovoltaic water pumping systems (PVWPSs) can provide a sustainable and cost-effective solution to improve access to domestic water and irrigation [13]. These systems can be classified into grid-connected and stand-alone PVWPSs based on their design [26,27]. Stand-alone PVWPSs can be configured as simple photovoltaic (PV) arrays that power water pumps directly during daylight hours [28] or as hybrid systems that integrate solar energy with other sources, such as diesel generators [29]. In some cases, storage solutions like batteries or water tanks may be added to store energy or water for use during periods of low sunlight [30].

Motor-pumps in PVWPSs can run on either DC or AC power, requiring a DC-AC converter in the latter case [31]. The type of motor-pump also varies depending on the water source [32]: surface pumps are typically used for drawing water from streams, rivers, or shallow groundwater (less than 7 m deep), while submersible pumps access deeper groundwater, which is more resilient to climatic variability and less prone to surface pollution [33,34]. For groundwater abstraction, boreholes are drilled to access the resource.

With Africa's high photovoltaic and groundwater potential [35,36], PVWPSs equipped with submersible pumps are increasingly seen as a viable solution for addressing water access challenges in rural areas [37]. Advances in PV efficiency and falling PV module costs have made PVWPSs more economically competitive [38,39]. Moreover, recent technical advancements in solar energy, including innovations in photovoltaic materials (e.g., perovskite [40] and organic [41] photovoltaic cells), improvements in solar system cooling methods [42], and the development of bifacial photovoltaic modules [43], continue to enhance the competitiveness of solar energy. Nevertheless, the proper selection and sizing of system components remains critical for ensuring stable and efficient operation, prompting extensive research into optimal system design. For instance, Kazem et al. explored the technical and economic feasibility of a photovoltaic water pumping system in rural Oman, optimizing the system using HOMER software to determine the size of the PV array, inverter, and battery storage, resulting in an energy cost of 0.309 USD/kWh, making it a competitive alternative to diesel-powered systems [30]. Study [21] aimed to minimize the life cycle cost and maximize the socio-economic impact of a PVWPS by optimizing the motor-pump selection, the water tank volume, the size of the PV array, and the system's location in the village. In a different approach, IWMI focused on appropriately sizing the motor-pump, determining its depth, and calculating the necessary installed PV power to ensure that the groundwater demand for irrigation is met all year long [32]. Although many studies, such as those mentioned above, focus on the optimal initial design of PVWPS, it is also important to address the durability of PVWPS components, particularly the borehole, a critical part of the system where water extraction occurs. This aspect will be the focus of the following sections of this literature review.

### 2.2 Aquifer and borehole response to pumping

**Fig. 1:**
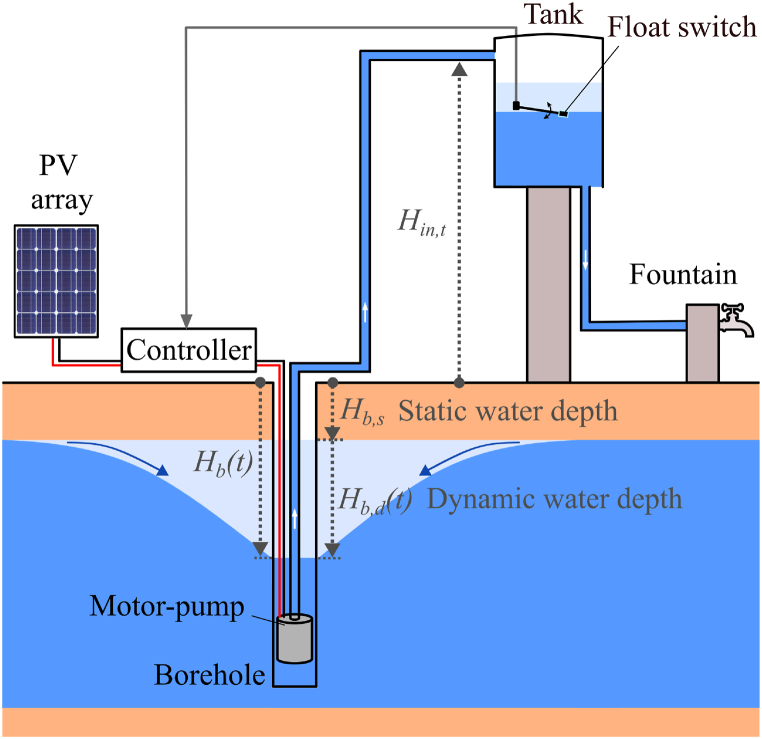
Photovoltaic water pumping system architecture for domestic water access; the water heights $H_{in,t}$, $H_{b}$, $H_{b,s}$ and $H_{b,d}$ are defined as positive.

Fig. 1 illustrates the standard architecture of a photovoltaic water pumping system (PVWPS) for domestic water use [44,45] and the aquifer's response to pumping [46]. The PV modules power a motor-pump, which transfers water from the borehole into a storage tank. Residents collect water at the fountain. A controller starts and stops the motor-pump depending on the water level in the tank, monitored by a float switch. When the motor-pump is idle, the water depth in the borehole, $H_{b}$, will gradually return to the static water depth, $H_{b,s}$. During the motor-pump operation, a cone of depression forms and there is a drawdown $H_{b,d}$ in the borehole (see Fig. 1) [46,47]. Higher flow rates result in increased drawdown $H_{b,d}$ and, consequently, an increase of the water depth in the borehole $H_{b}$. The physical reference for the water depths $H_{b},H_{b,s}$ is the ground level. The physical reference for the drawdown $H_{b,d}$ is the static water depth (see Fig. 1).

The water depth in the borehole $H_{b}$ can be expressed as [18,19]:

(1) $$H_{b}\left(t\right)=H_{b,s}+H_{b,d}\left(t\right)$$

with

(2) $$H_{b,d}\left(t\right)=\sum_{n=0}^{N}\kappa_{n}Q_{p}\left(t-n\;\Delta T\right)+\sum_{n=0}^{N}\mu_{n}Q_{p}{\left(t-n\;\Delta T\right)}^{2}$$

$Q_{p}$ is the pumped flow rate, $\kappa_{n}$ and $\mu_{n}$ are positive fitting coefficients and ΔT is the characteristic response time of the borehole. N is a fitting parameter that represents the effect of the past flows on the borehole water depth. Its value is chosen based on available data using valid statistical methods [48]. The parameters N, ΔT, $\kappa_{n}$ and $\mu_{n}$ can be obtained by identification from measurements of the pumped flow rate $Q_{p}$ and of the corresponding borehole water depth $H_{b}$. The water heights $H_{b,s}$ and $H_{b,d}$ are defined as positive. It is important to note that $H_{b,s}$, N, ΔT, $\kappa_{n}$ and $\mu_{n}$ (thus $H_{b,d}$) usually vary along the lifetime of the PVWPS [18,49], i.e., the value of the parameters at the PVWPS installation may differ from their value after a few months/years of operation of the system. It should be noted that equations (1), (2) are a generalization of Jacob's equation [50] and are data-driven. Other equations, such as the Theis equation, can also be used to express the water depth in the borehole. These alternative equations can replace equations (1), (2) without altering the overall approach presented in this article.

### 2.3 Standard pumping tests

#### 2.3.1 Principle

Data is collected through pumping tests to monitor the impact of pumping on groundwater resources. Two types of pumping tests can be distinguished: (1) those which provide information solely on the hydraulic characteristics of the borehole and (2) those which provide information on the hydraulic characteristics of both the borehole and the aquifer. For the first type, water is extracted from the borehole at given flow rates $Q_{p}$ and the water depth in the borehole $H_{b}$ is monitored [18,19]. For the second type, both the water depth in the borehole $H_{b}$ and the water depth in observation wells is monitored [46,51]. Observation wells are small diameter boreholes drilled some meters away from the borehole of interest [52]. The objective of observation wells is to estimate certain aquifer parameters (e.g., transmissivity and storativity) by measuring the water depth evolution inside them during the pumping test and to compare it with the water depth evolution inside the borehole of interest. Even though these latter tests provide information on the aquifer, they are also significantly more expensive notably because they incur extra costs due to drilling the observation wells [53,54]. Given the frequently challenging economic conditions in rural areas of developing regions, this study focuses on tests that provide information solely on the borehole, i.e., without observations wells, as it is often the case for this type of installations [55].

Thus, the considered pumping tests consist in pumping water from a borehole and measuring the water depth in the borehole $H_{b}$ over time. “Multiple step drawdown tests” are the most common tests. The water is pumped at a constant flow rate for several steps, each lasting about 1 h, with variations in the flow rate between each step [19,56]. A couple of steps (typically three or four) are usually sufficient to acquire the desired information, and it is often what is done in practice [18,57]. A recovery phase (no pumping) of the water depth of around 1 h may be implemented between each pumping step [58]. “Long-term pumping tests” can also be done and are complementary to multiple step drawdown tests [59]. For long-term tests, the water is pumped at a constant rate, over a long period of time (often more than a day). A recovery phase, which can last several hours or even several days, is implemented in the case of long-term pumping tests.

**Fig. 2:**
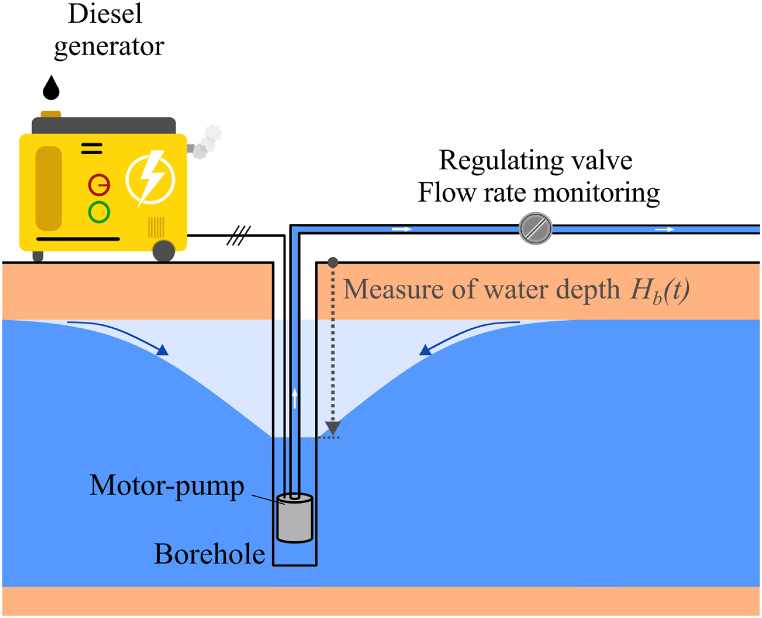
Set-up for standard pumping tests.

Even though they are usually done right after the borehole drilling or before the borehole recommissioning, pumping tests can in theory be done at any time throughout the lifetime of the borehole. Current tests require a regulated pumping flow rate $Q_{p}$, meaning that the motor-pump being used must have a dispatchable and sufficient energy source to power the motor-pump at will, usually a diesel generator. Fig. 2 shows the typical set-up used for performing standard pumping tests. It is also important to note that pumping tests have not evolved much for more than 50 years, from Ref. [19] (in 1963) to Ref. [53] (in 2019).

#### 2.3.2 Cost

Pumping tests represent a non-negligible cost as they can last hours, even days, and require human and material resources [21,60]. In developing regions, it is difficult to find accurate information on the cost of pumping tests because it is usually included in the drilling cost of the borehole. The pumping tests cost includes the following elements: equipment for measuring the water depth in the borehole (e.g., hydrostatic pressure sensor) [61]; data logger, or a notebook to manually log the data; diesel generator and fuel as well as manpower to carry out the test, supervise the data collection and analyse the results throughout the test. Costs tend to increase when the borehole location is far away from the office of the company doing the test, due to higher travel expenses [62,63].

### 2.4 Role of pumping tests in the legislative framework on borehole management

There is no clear legislative framework for an ideal management to ensure borehole sustainability. However, several regulations can be identified that are currently implemented for the different stages of a pumping system's lifetime.

Before the installation of a pumping system on the borehole or at the borehole recommissioning, numerous countries all around the world, including countries in sub-Saharan Africa, require pumping tests to be done [64,65]. In the case of sub-Saharan Africa, these pumping tests are in most cases a multiple step drawdown test of several hours (with three/four steps, including recovery), followed by a long-term pumping test of a few days (including recovery) [63]. A key objective is to determine a “safe yield”, i.e., a maximum pumping flow rate, that will ensure a certain sustainability for the borehole use [65,66]. Another objective can be to model the borehole behaviour, e.g., identify the value of the parameters $H_{b,s}$, N, ΔT, $\kappa_{n}$, $\mu_{n}$ from the measurements of $Q_{p}$ and $H_{b}$ (see equations (1), (2)). Modelling the borehole behaviour helps to predict the performance of the installed pumping system and make sure that the users’ water requirements are met in a sustainable manner [18].

After the installation of a pumping system, certain countries have legislation to protect the borehole over time [67]. Most of the focus is on water contamination, with regulations on minimum distances between the borehole head and other facilities, on the borehole cap, on the gradient of the slope around the borehole head to prevent surface water from entering the borehole, and on the presence of waste near the borehole [63,68]. Certain countries have developed systems to keep track of existing boreholes (e.g., boreholes must have identification plates and records have to be kept [68]). Recent recommendations encourage the monitoring of boreholes over their lifetime [69]. For instance, in Mali and Senegal, governments adopted plans to study groundwater more in depth and monitor quantitatively groundwater resources and their evolution [70,71].

### 2.5 Novel contribution of the article

It is observed that, despite recent recommendations, pumping tests are typically conducted only after borehole drilling or at the borehole recommissioning. This limitation is primarily due to the substantial operations and costs associated with performing pumping tests throughout the lifespan of the pumping system. For instance, for PVWPS, performing a standard pumping test with a diesel generator involves carrying a diesel generator to the site, disconnecting the photovoltaic array, opening the borehole, and then restarting the entire PVWPS after having performed the tests. In addition, such tests prevent users from fetching water during the test and incur substantial diesel consumption costs. The lack of regular pumping tests during the lifetime of PVWPS hinders effective monitoring of the boreholes’ condition over time, failing to account for the long-term effect of pumping on their health [65,69].

To address these issues, this work presents a novel method for facilitating continuous borehole monitoring in PVWPS: photovoltaic pumping tests. This innovative approach leverages the PVWPS's own photovoltaic modules as the energy source for conducting pumping tests, thereby eliminating the need for diesel generators. This method not only simplifies the monitoring process compared to standard pumping tests but also reduces operational costs, emissions and logistical complexities. Moreover, it allows for uninterrupted water supply to users during testing, a significant advantage over standard methods. To the best of the authors' knowledge, no prior studies have proposed such a method for evaluating the evolution of boreholes throughout the lifespan of PVWPS. This article thus introduces a promising tool that can contribute to the long-term sustainability of photovoltaic water pumping systems, potentially benefiting stakeholders involved in water access and management in rural areas.

## 3 Photovoltaic pumping tests

In Section 3.1, the objective of photovoltaic pumping tests, which aim to monitor borehole water levels at various flow rates without disrupting the operation of the PVWPS, is outlined. A detailed procedure for conducting these tests, including the required equipment and manpower, is provided in Section 3.2. A new indicator, the Borehole Dynamic Health Indicator (BDHI), is introduced in Section 3.3 to evaluate borehole health over time. Finally, in Section 3.4, the costs and benefits of the proposed novel testing method are discussed, demonstrating its potential to improve borehole maintenance, prevent costly system failures, and enhance the sustainability of PVWPS.

### 3.1 Objective

The objective of photovoltaic pumping tests is to measure the water depth in the borehole for various pumping flow rates, after the installation of the PVWPS, without interfering with the PVWPS operation (the users must maintain access to the pumped water throughout the test) and at a reduced cost. Such tests can first help to identify potential problems at the borehole level. For instance, if from a test at a moment in time to another a few months/years later, the drawdown significantly increases for the same pumped flow rate, it could indicate that the borehole should be unclogged (using acids, polyphosphates or chlorine [59]). These tests also enable updating the borehole parameters $H_{b,s}$, N, ΔT, $\kappa_{n}$ and $\mu_{n}$, which notably permit more accurate prediction of system performance [18]. Through this section, the procedure for photovoltaic pumping tests (including equipment, protocol and human resources) is detailed. Additionally, a new indicator – referred to as the borehole dynamic health indicator – is introduced to assess the state of health of the borehole over time and the costs and benefits of such tests are quantified.

It should be noted that, for the sake of conciseness, this article primarily refers to the use of photovoltaic pumping tests for PVWPS in domestic water application and a case study for such a PVWPS is presented. However, such tests are also applicable to PVWPS for irrigation. The only small difference would be that these systems do not necessarily include a water tank. In this case, the flow rate sensor (see Section 3.2.1) can be placed next to the output of the borehole and the height between the ground and the water tank entry $H_{in,t}$ (see Fig. 1 and Section 3.3) can be taken equal to the height between the ground at the level of the borehole and the field height (typically less than a few meters).

### 3.2 Implementation

#### 3.2.1 Equipment required

**Fig. 3:**
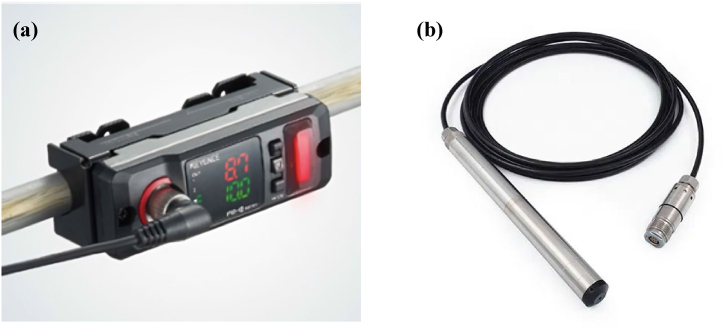
Example of (a) flow rate sensor [73] and (b) hydrostatic pressure sensor [72].

Two quantities need to be measured: the pumped flow rate $Q_{p}$ and the water depth in the borehole $H_{b}$. The main proposed solution for measuring $Q_{p}$ is to use a clip-on flow sensor (see Fig. 3a), which is attached around the pipe between the motor-pump and the tank, and uses sound waves to measure the flow going through it. The clip-on flow sensor is installed before the pumping test and removed afterwards without interfering with the PVWPS operation. It is therefore considered ‘non-intrusive’. To measure $H_{b}$, a hydrostatic pressure sensor, such as the DCX-22 SG [72], can be used (see Fig. 3b). The sensor is positioned below the water level and measures the hydrostatic pressure induced by the height of water above it. In general, the hydrostatic pressure reading is automatically recorded by the sensor. In some cases, $Q_{p}$ and $H_{b}$ could be measured differently. For example, a turbine flow sensor installed on the pipe between the motor-pump and the tank may be used for the measurement of $Q_{p}$. However, the turbine flow sensor should be set up during the PVWPS installation and always remains on-site, which may be less practical depending on the specific case.

#### 3.2.2 Protocol

**Fig. 4:**
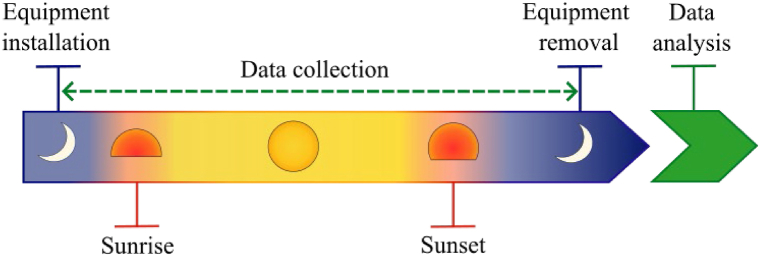
Timeline of the photovoltaic pumping test.

The protocol timeline is proposed and summarized in Fig. 4. The installation of the equipment is carried out approximately 1 h before sunrise to be ready to start the measurements when the water in the borehole is still at its static depth (no pumping during the night). The data collection starts 30 min before sunrise. The measurement is stopped ∼2–3 h after sunset, in order to collect data throughout the whole day, and to observe the recovery of the water level without pumping. Finally, the equipment is removed, and the data can be analysed. Every 30 min, the technician verifies that the data is being logged correctly. A time step between 1 and 15 min is recommended to acquire the data. In case of the absence of an automatic data logger, the data could be collected manually by the technician simply by writing down the data from the water depth and the flow sensor every 15–30 min.

This test should be performed relatively regularly as it is used to detect any abnormal evolution of the borehole that could affect the PVWPS's lifespan and ability to meet its requirements. Conducting the test annually or every two years should be sufficient, unless there is any sudden sign of weakness, such as a noticeable loss of pressure or flow in the water coming out of the borehole. If possible, it is advised to perform the test during a period when the water in the borehole is the deepest, often by the end of the dry season [41], and to reproduce it at this same period from one year to the other. Indeed, one significant threat for the PVWPS sustainability is if the water level in the borehole is too low [74].

#### 3.2.3 Manpower

As there are only two sensors to install to perform the photovoltaic pumping test, one technician is sufficient to set up the equipment and conduct the test. This person will oversee the installation of the equipment, launch the data collection, verify regularly that the sensors function properly and remove the equipment at the end of the test. They will remain on-site at the PVWPS location during the test duration. This person should have basic theoretical knowledge or have completed a training course on boreholes, pumping tests as well as flow rate and water level monitoring. They will also need to know how to install the equipment, to be able to diagnose a malfunction, and to fix the sensors when they are not working properly. It is envisaged that this person could become a specialist in carrying out photovoltaic pumping tests and could undertake several photovoltaic pumping tests in a given region. If the person performing the test is not from the same company(ies) as the one(s) which drilled the borehole and installed the motor-pump, they should obtain relevant information about the site from these parties (e.g., borehole depth and diameter, motor-pump depth).

### 3.3 Borehole dynamic health indicator (BDHI)

In order to interpret the measurements from the photovoltaic pumping test, the borehole dynamic health indicator BDHI is introduced, which is expressed as follows:

(3) $$BDHI=\frac{H_{b,d,sim\;}\left(\tilde{Q_{p}}\right)}{H_{b,d,0,sim}\left(\tilde{Q_{p}}\right)}$$

where $H_{b,d,sim}\left(\tilde{Q_{p}}\right)$ is the value of the drawdown $H_{b,d}$ (see equation (2)) determined from the borehole parameters N, ΔT, $\kappa_{n}$ and $\mu_{n}$ identified through the photovoltaic pumping test. $H_{b,d,0,sim}\left(\tilde{Q_{p}}\right)$ is the drawdown $H_{b,d}$ determined from the initial borehole parameters N, ΔT, $\kappa_{n}$ and $\mu_{n}$ identified during the standard pumping test carried out at the PVWPS installation. Both drawdown values are simulated using the characteristic pumping flow rate value $\tilde{Q_{p}}$, defined by:

(4) $$\tilde{Q_{p}}=\frac{P_{p}\cdot \eta_{mp}}{\rho \cdot g\cdot \left(H_{b,s,0}+H_{in,t}\right)\;}$$

where $P_{p}$ is the peak power of the PV modules under standard test conditions (STC), $\eta_{mp}$ is the motor-pump efficiency, ρ is the water density (1000 kg m^−3^) and g is the gravitational acceleration (9.81 m s^−2^). $H_{b,s,0}$ is the initial static water depth in the borehole determined during the standard pumping test at installation and $H_{in,t}$ is the height between the ground level and the level at which water enters the tank (see Fig. 1). $\left(H_{b,s,0}+H_{in,t}\right)$ thus corresponds to a static head. For the sake of simplicity, for computing $\tilde{Q_{p}}$, the value of 0.35 can be considered for $\eta_{mp}$, as an approximation [75]. $\tilde{Q_{p}}$ corresponds to a characteristic flow rate for the system under investigation.

The borehole dynamic health indicator BDHI is used to evaluate the evolution of the drawdown relative to the initial state of the borehole. As it focuses on the drawdown, this indicator is complementary to the measure of $H_{b,s}$, which is also obtained from the photovoltaic pumping tests.

### 3.4 Cost and benefits

To discuss its feasibility, the cost $C_{tot}$ of a single photovoltaic pumping test is estimated:

(5) $$C_{tot}=C_{manpower}+C_{equipment}+C_{transport}$$

where $C_{manpower}$ is the cost of the manpower, $C_{equipment}$ is the cost of the equipment, and $C_{transport}$ is the cost of the transport. The manpower cost is estimated from the local minimum hourly wage $S_{h}$ in the country, to which a 20 % tax is added, and multiplied by the test duration h (in hours). This cost is multiplied by 3 to cover other expenses and make the job attractive in terms of salary:

(6) $$C_{manpower}=3\cdot h\cdot \left(1.2\cdot S_{h}\right)$$

To compute the equipment cost, it is considered that the equipment is used 200 times before replacement. The cost of equipment per test is:

(7) $$C_{equipment}=\left(C_{sensor}/200\right)$$

where $C_{sensor}$ is the sum of the price of all sensors used. Finally, the cost of the transport with a vehicle $C_{transport}$ depends on the location of the PVWPS and is estimated as follows:

(8) $$C_{transport}=2\cdot d\cdot Co_{diesel}\cdot C_{diesel}$$

where d is the distance to be travelled from the closest urban area (>100 000 inhabitants) to the location of the PVWPS (km), $Co_{diesel}$ is the diesel consumption of the vehicle (L/km) and $C_{diesel}$ is the average diesel cost for the country (€/L).

Even though photovoltaic pumping tests have associated costs, they are beneficial for the sustainability of the PVWPS. In particular, they can help diagnose a significant increase of the water depth in the borehole $H_{b}$, which may result from poor borehole maintenance, overextraction of the PVWPS, or external factors such as overextraction of pumping systems installed on the same aquifer [76]. Diagnosing this too important increase of $H_{b}$ thanks to photovoltaic pumping tests can enable the implementation of appropriate measures to preserve the sustainability of the PVWPS and possibly of the aquifer. Examples of measures include unclogging the borehole; adapting the use of the PVWPS in an organized way (e.g., capping the amount of water that can be collected at the PVWPS); alerting and discussing with neighbouring pumping systems managers and possibly with local governments and experts.

In addition to threatening the sustainability of the PVWPS and of the aquifer, failure to diagnose a significant increase of $H_{b}$ and to enforce adapted measures can have technical consequences that can directly impact the cost of the PVWPS. The first possible consequence is that, if $H_{b}$ increases too much, then the total dynamic head (sum of $H_{b}$, $H_{in,t}$ and of the pipe losses) can become too high for the installed motor-pump [77]. This would reduce and may even eliminate the pumped flow rate. A second possible consequence of a significant increase of $H_{b}$ is that the water level may drop below the motor-pump, which may keep running without water to cool it down. This overheating could result in the failure of the motor-pump [78]. Even if the motor-pump has protection and stops, this will reduce the pumped flow rate [75] and may shorten the motor-pump's lifespan [79]. As a result of these two consequences, several costs can incur. First, the motor-pump may have to be replaced (e.g., by one of higher maximum pumping height). Second, the borehole may have to be drilled deeper to place the motor-pump deeper. Third, the borehole and even the PVWPS itself may be abandoned by the inhabitants because they do not fulfil their needs.

## 4 Case study

The results and costs of standard and photovoltaic pumping tests are here presented and discussed for a PVWPS installed in a village in Burkina Faso. More specifically, the characteristics of the considered PVWPS are described in Section 4.1. The results of the standard and photovoltaic pumping tests are provided in Sections 4.2, 4.3 respectively. Then, in Section 4.4, the benefits in terms of borehole characterization, groundwater sustainability, and financial viability of performing photovoltaic pumping tests in addition to the initial standard pumping tests are quantified for the considered case.

### 4.1 Description of the PVWPS of the village of Gogma in Burkina Faso

**Fig. 5:**
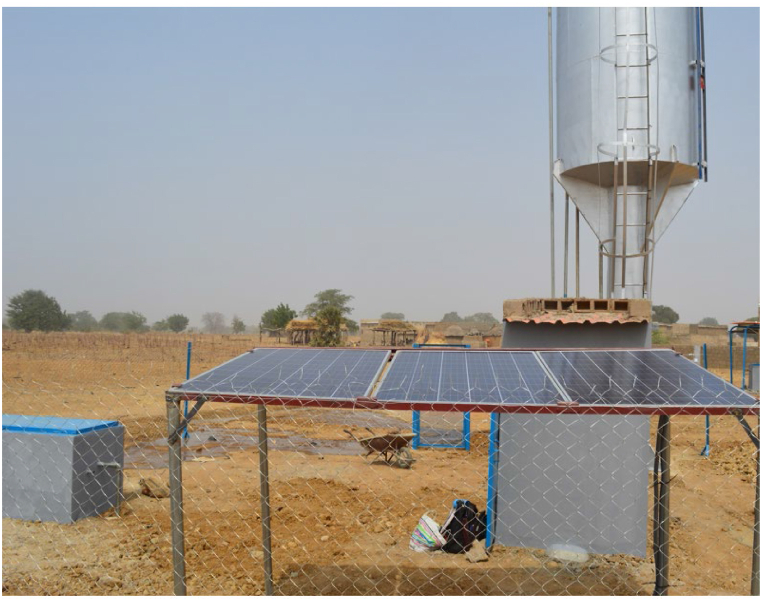
Picture of the PVWPS of Gogma.

Gogma is a village situated in the central-eastern region of Burkina Faso, in Sub-Saharan Africa, with a population of 1100 residents. There is no centralized water supply network in Gogma. The village experiences a tropical savanna climate with distinct wet and dry seasons. The wet season, from June to September, brings most of the annual rainfall, averaging between 700 and 900 mm, while the dry season, from October to May, is characterized by high temperatures often exceeding 40 °C, along with minimal rainfall [80]. These climatic conditions, along with dry and dusty Harmattan winds from the Sahara, contribute to water scarcity, making sustainable water management critical. To address this, a PVWPS has been installed in Gogma in January 2018 (see Fig. 5). An informative video of the system is available at: https://youtu.be/VrjM0edKVsI. The PV array has a peak power $P_{p}$ of 620 W_p_, the motor-pump is a SQFlex 5A-7 [81] and the tank volume is 11.4 m^3^. This PVWPS serves approximately 280 village inhabitants, meeting a daily water demand of around 7 m^3^. The borehole is 56 m deep, has an interior diameter of 0.11 m and the motor-pump is at a depth of 30 m. The height between the ground level and the level at which the water enters the tank $H_{in,t}$ is 7.6 m.

### 4.2 Standard pumping test

**Fig. 6:**
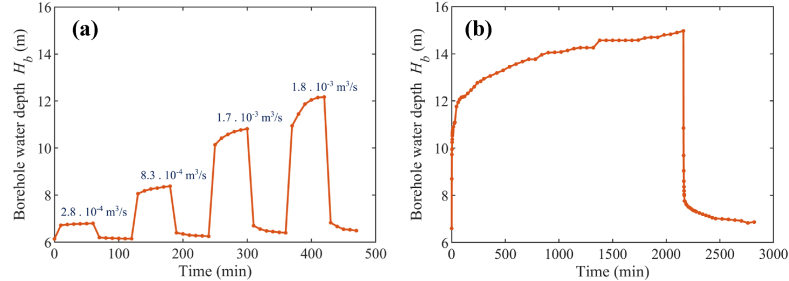
Measured water depth in the borehole during (a) the multiple step drawdown tests and (b) the long-term pumping test (pump flow rate: 1.8 ·10^−3^ m^3^/s).

Two types of pumping tests were conducted at the PVWPS following the borehole drilling (in November 2017), by the company Sogedaf, headquartered in Burkina Faso. The first was a multiple step drawdown test performed on November 5, 2017, for 4 distinct flow rates (2.8·10^−4^ m^3^/s, 8.3·10^−4^ m^3^/s, 1.7·10^−3^ m^3^/s and 1.8·10^−3^ m^3^/s). Each step of entailed continuous monitoring of the borehole water depth $H_{b}$ for 1 h during pumping at the specified flow rate, followed by an additional hour without pumping to observe the recovery phase. The results are displayed in Fig. 6a. The second test, a long-term pumping test, was executed from November 8 to November 9, 2017. During this test, the borehole's water depth was monitored over a 36-h period while pumping at a constant flow rate of 1.8·10^−3^ m^3^/s and for an additional 11 h without pumping to study the recovery phase. These tests required significant logistics (including human resources, equipment, and sensors), which may introduce uncertainties, delays, and additional costs. The results are shown in Fig. 6b. It is observed from these pumping tests that the static water depth $H_{b,s}$, i.e., the stabilized water depth in the borehole when there is no pumping, is equal to 6 m at this time of the year. The increase of $H_{b}$ from this static water depth while pumping corresponds to the dynamic behaviour of the water depth in the borehole.

### 4.3 Photovoltaic pumping tests

**Fig. 7:**
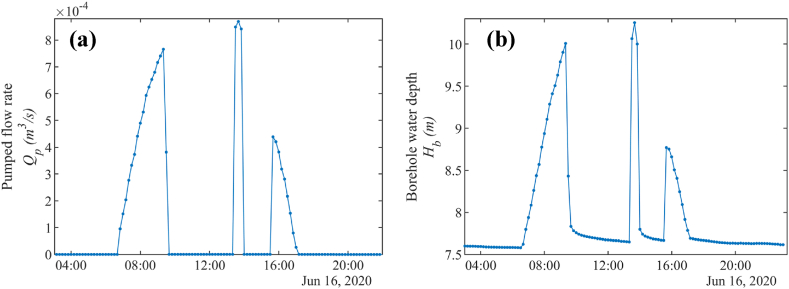
Measurements of (a) flow rate and (b) water depth in the borehole during a photovoltaic pumping test (June 16^th^, 2020).

**Fig. 8:**
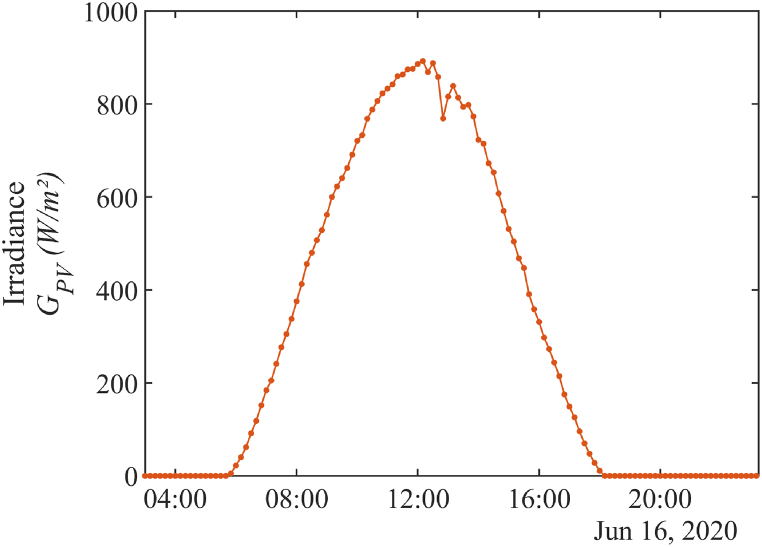
Measurement of irradiance on the plane of the photovoltaic modules $G_{PV}$ during a photovoltaic pumping test (June 16^th^, 2020).

For measuring the water depth in the borehole, a DCX-22 SG hydrostatic pressure sensor (see Fig. 3b) has been installed [72]. The water depth in the borehole is measured with a time step of 10 min. For measuring the pumped flow rate, a YF-DN40 turbine flow sensor has been installed [82] connected to a custom data logger [21]. Water flow rate measurements with a 10 min time step are used. In the case study, a turbine flow meter was utilized to measure the flow rate $Q_{p}$ as this PVWPS has been monitored since its installation, notably for scientific research purposes. However, the use of a clip-on flow sensor is still recommended for any usual photovoltaic pumping test. Fig. 7 illustrates the measurements done during a photovoltaic pumping test on the June 16, 2020. The behavior of the flow rate and the variation in water depth in the borehole closely mirrors the pattern of irradiance (see Fig. 8), as both are directly dependent on the available solar power (see Section 2.2). The only significant difference is the interruptions in pumping, which impact the flow rate and water depth in the borehole. These interruptions occur when the water tank is full.

### 4.4 Quantifying the costs and benefits from photovoltaic pumping tests

#### 4.4.1 Improved borehole characterization

The objective of this Section is to quantify the interest of performing photovoltaic pumping tests in terms of borehole characterization for the considered case study. For this purpose, the datasets considered are presented in Table 1, which include data from standard pumping test (the multiple step drawdown test in particular) and photovoltaic pumping tests over five years. Regarding photovoltaic pumping tests, there are two datasets from the end of the dry season (Photovoltaic Pumping Tests I and III) and two datasets from the wet season (Photovoltaic Pumping Tests II and IV).

**Table 1:** Results for the identification of the borehole model coefficients for the various datasets.

Test	Multiple step drawdown test	Photovoltaic Pumping Test I	Photovoltaic Pumping Test II	Photovoltaic Pumping Test III	Photovoltaic Pumping Test IV
**Date (i.e., "identification period")**	Nov 05, 2017	Jun 11, 2019	Nov 16, 2019	Jun 10, 2020	Sep 22, 2020
	*Wet season*	*Dry season*	*Wet season*	*Dry season*	*Wet season*
**Validation period**	N/A	12–24 Jun 2019	17–29 Nov 2019	11–23 Jun 2020	23 Sep - Oct 06, 2020
$H_{b,s}$**(m)**	6	7.8	6.0	7.6	5.9
$\kappa_{0}$**(m**^**−**^**^2^.s)**	2.0 ·10^3^	2.3 ·10^3^	2.3 ·10^3^	2.3 ·10^3^	2.4 ·10^3^
$\mu_{0}$**(m**^**−**^**^5^.s**^**2**^**)**	5.8 ·10^5^	7.2 ·10^5^	4.4 ·10^5^	7.0 ·10^5^	3.0 ·10^5^
$R^{2}$**of the identification**	0.97	0.99	0.99	0.99	0.99
NRMSE**on**$H_{b}$**when using the values of**$H_{b,s}$**,**$\kappa_{0}$**and**$\mu_{0}$**obtained from the identification period (%)**	N/A	1.8	2.0	1.6	4.9
NRMSE**on**$H_{b}$**when using the values of**$H_{b,s}$**,**$\kappa_{0}$**and**$\mu_{0}$**obtained from the multiple step drawdown test (%)**	N/A	23.5	2.3	21.5	6.5
$H_{b,d,sim\;}\left(\tilde{Q_{p}}\right)$**(m)**	4.7	5.5	4.8	5.5	4.6
BDHI	1	1.18	1.03	1.17	0.99

For this case study, a borehole water depth model (see equations (1), (2)) with *N* = 0 is found to be sufficient for predicting the borehole water depth $H_{b}$ with adequate accuracy. Additional parameters (for N>0) have low statistical significance [18]. For each dataset, the identification of the three parameters of the borehole model $\left(H_{b,s},\kappa_{0},\mu_{0}\right)$ is performed by using measured values of the pumped flow rare $Q_{p}$ and of the borehole water depth $H_{b}$ for a “identification period” dataset. This dataset corresponds to either the multiple step drawdown test or to a photovoltaic pumping test (see Table 1).

The value of these parameters is used to simulate the borehole water depth $H_{b}$ for the flow rates $Q_{p}$ of the “validation period” dataset. For the same validation period, $H_{b}$ is also simulated using the values of the borehole model parameters obtained from the standard pumping tests. Then, to quantify the interest of performing photovoltaic pumping tests to better estimate $H_{b}$, for each set of borehole model parameters, the error between the simulated and the measured water depth is evaluated for the validation period. This error is computed through the normalized root mean square error NRMSE:

(9) $$NRMSE=\sqrt{\frac{\sum_{i=1}^{n}{\left(H_{b,meas}\left(i\right)-H_{b,sim}\left(i\right)\right)}^{2}}{\sum_{i=1}^{n}H_{b,meas}{\left(i\right)}^{2}}}$$

where n is the number of points in the validation period, $H_{b,meas}$ and $H_{b,sim}$ are respectively the measured and the simulated water depth for the validation period. The calculation of the NRMSE is interesting to highlight the interest of this type of test in this research-oriented case study but is not essential for the protocol's application. The results are provided in Table 1.

Furthermore, the borehole dynamic health indicator BDHI is computed using the design parameters of the PVWPS of Gogma ($P_{p}$ = 620 W_p_, $\eta_{mp}$ = 0.35, $H_{b,s,0}$ = 6 m, $H_{in,t}$ = 7.6 m). Thus, a characteristic pumping flow rate $\tilde{Q_{p}}$ (see equation (4)) is obtained, equal to 1.6 ·10^−3^ m^3^ s^−1^. Given the initial borehole parameters $\kappa_{0\;}$ and $\mu_{0}$ from the initial multiple step drawdown test (see Table 1) and through equation (2), $H_{b,d,0,sim}\left(\tilde{Q_{p}}\right)$ is obtained and equals to 4.7 m. The BDHI (see equation (3)) is then computed for each photovoltaic pumping test and the results are provided in Table 1.

The $R^{2}$ values for photovoltaic pumping tests (average $R^{2}$ of 0.99) highlight their high accuracy in determining the borehole parameters ($H_{b,s}$, $\kappa_{0}$, $\mu_{0}$). This level of accuracy is comparable to that of standard pumping tests conducted for the PVWPS in Gogma, which achieved an R^2^ of 0.97. Moreover, the precision of the photovoltaic pumping tests in determining borehole parameters can be compared to that achieved by standard pumping tests in other studies, where R^2^ values are found to typically range between 0.95 and 1.00 [83,84]. Thus, the accuracy of photovoltaic pumping tests for determining borehole parameters is equivalent with that of standard pumping tests. However, photovoltaic pumping tests are more cost-effective, require less logistical support, and allow for uninterrupted water supply during testing compared to standard pumping tests (see Sections 2.5, 4.4.2).

The Normalized Root Mean Square Error (NRMSE) for each set of borehole parameters obtained from the identification period illustrates the accuracy of the model of the borehole water depth $H_{b}$ (see equation (1) and (2)). Moreover, results indicate that the performance on the borehole water depth estimation over the validation period is improved when recent borehole parameters are used (average NRMSE of 2.8 %) in comparison with the estimation using initial standard pumping test parameters (average NRMSE of 14.6 %). These good results in determining borehole parameter values and estimating borehole water depth indicate that selecting a 10-min time step (see Section 4.3) for measurements during the photovoltaic pumping tests is appropriate.

It is observed that the static water depth $H_{b,s}$, the simulated drawdown $H_{b,d,sim\;}\left(\tilde{Q_{p}}\right)$ and the borehole dynamic health indicator BDHI do not strongly vary across all tests thus indicating the good state of health of the borehole, certainly due to an adequate borehole maintenance and PVWPS operation over time. It is also interesting to notice that the slight variation of these indicators seems related to seasonality and that these indicators reach their highest value for the dry season. In the case of the PVWPS installed in Gogma (where the motor-pump is positioned at a depth of 30 m), the static water depth $H_{b,s}$ values assessed through the photovoltaic pumping tests, indicate that even during extended pumping periods or dry conditions, the motor-pump would not be at risk. Even though a test each year or every two years is proposed in Section 3 for practicality reasons and is deemed sufficient in most cases, it can be interesting, if it is financially and practically possible, to carry out more than one test for a given year to learn about the impact of seasonality.

**Fig. 9:**
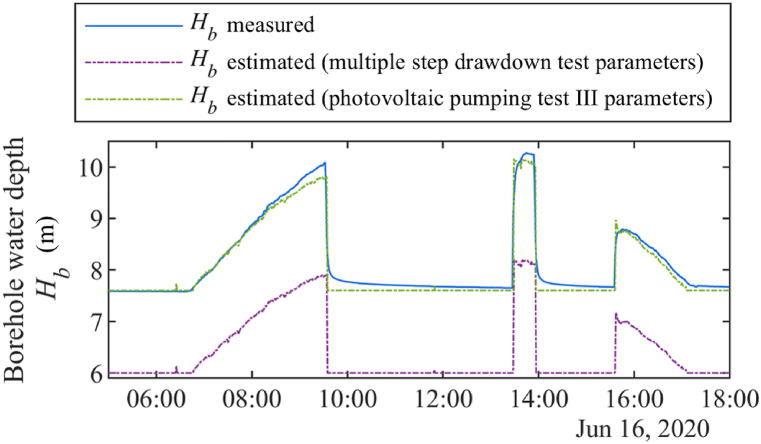
Measured and estimated borehole water depth $H_{b}$. The estimations are made based on different borehole parameters.

As highlighted in the previous paragraph, photovoltaic pumping tests allow to update accurately the values of the borehole parameters and to determine the state of health of the borehole through the evolution of the static water depth $H_{b,s}$ and of the indicator BDHI. They also permit to estimate more accurately the borehole depth $H_{b}$ for a specific period, which is important to simulate the PVWPS operation [18]. In Fig. 9, the measured borehole water depth $H_{b}$ on the June 16, 2020 (which belongs to the validation period of the photovoltaic pumping test III) is plotted, along with its estimation using either borehole parameters from the multiple step drawdown tests or from the photovoltaic pumping tests III. It is observed that the estimation of the water depth is more accurate when using more recent parameters (those of the photovoltaic pumping test III). Additionally, it is noted that $H_{b,s}$ has a significant impact on the estimation of $H_{b}$ in this case.

#### 4.4.2 Economic analysis

Regarding the standard pumping test for the case of Gogma, the multiple step drawdown test costs $511 and the long pumping test costs $2050. In comparison, by applying equations (5), (6), (7), (8) for the case of Gogma, the cost of performing one photovoltaic pumping test in Gogma is estimated equal to $43. It is evenly split between manpower, equipment and transport. This cost estimation is obtained assuming that a FD-Q32C clip-on flowmeter [73] and a DCX-22 FG hydrostatic pressure sensor [72] are used and that the test is conducted by a technician coming from Ouagadougou, Burkina Faso's capital.

Besides, as mentioned in Section 3.4, performing photovoltaic pumping tests can yield important cost savings over the lifetime of the system such as not having to replace the motor-pump prematurely and not having to drill a new borehole (if the PVWPS is abandoned for instance). For the case of Gogma, the cost of photovoltaic pumping tests ($43) is compared to the cost of a motor-pump and the estimated cost of drilling a new borehole. The motor-pump of Gogma costed $2200, thus it would take 51 photovoltaic pumping tests to add up to the cost of replacing the motor-pump in case it breaks down prematurely. The lifetime of the motor-pump is considered to be 10 years [21]. Over this time, the total cost of performing regular photovoltaic pumping tests on a two-years basis would add up to $215. This is less than 10 % of the cost of a new motor-pump if it had to be replaced prematurely. Drilling the borehole of Gogma costed $8300. This corresponds to the cost of performing 193 photovoltaic pumping tests. The lifetime of a borehole is considered to be 50 years [21]. Over this time, the total cost of carrying out photovoltaic pumping tests on a two-years basis would add up to $1075, which is 13 % of the cost of drilling a new borehole.

Over the life expectancy of both the motor-pump and the borehole, not only are photovoltaic pumping tests less expensive than the premature replacement of these components, but the cost is also spread evenly over time. This means that no large payment must be made at once, which would be the case if a major component of the PVWPS had to be replaced. Besides, in some cases, the failure of the PVWPS cannot be addressed by the local community (e.g., lack of sufficient financial funds for material replacement, installation time too long) and can lead to the abandonment of the system. On top of that, photovoltaic pumping tests are compatible with the normal use of the PVWPS, whereas changing a component of the PVWPS would render it unusable for the whole time before (it may take quite some time for the repairing company to come to the site) and during reparation.

## 5 Discussion

This study underscores not only the importance but, more crucially, the effectiveness of monitoring borehole performance throughout the lifecycle of Photovoltaic Water Pumping Systems (PVWPS). By detecting declines in PVWPS operation, the proposed method facilitates early identification of issues such as borehole clogging, a leading cause of borehole failure [84,85]. Clogging can also degrade the perception of water quality [86,87]. Implementing the monitoring of the borehole performance on a regular, systematic schedule would help plan preventive interventions, significantly reducing the risk of system failure and safeguarding communities from the need to revert to unsafe water sources [88]. This is particularly critical in rural or off-grid areas, where delays in obtaining spare parts due to long distances can exacerbate the consequences of system breakdowns [89].

An unhealthy borehole not only threatens water access but also diminishes the efficiency of PVWPS. For instance, a clogged system pumps less water for the same energy input [90], which is an unsustainable loss in regions like sub-Saharan Africa (SSA), where over 600 million people already lack access to electricity [91]. In such settings, maintaining high energy efficiency is important. Failures in PVWPS could also undermine the broader potential of these systems to address water and energy access gaps. Case studies in India demonstrate that a single malfunction can lead to widespread distrust in PVWPS, with farmers dissuading others from adopting the technology [92]. Thus, proactive diagnosis methods such as the one proposed in this article are vital to fostering confidence in the widespread adoption of improved water pumping systems, such as PVWPS.

Beyond the technical and efficiency concerns, PVWPS failures can have profound social implications, particularly with regard to gender inequalities [93]. Research shows that when PVWPS fail, the burden of collecting water from older, less efficient systems typically falls on women [93], even though water demand often increases after a PVWPS installation [94]. Preventing such failures is thus not only an issue of technical maintenance but also one of alleviating the disproportionate labour burden on women in these communities.

In addition to maintaining borehole functionality, the proposed monitoring approach also allows for the systematic measurement of static water depth. Regularly conducting such tests could contribute valuable data on groundwater levels across Africa—a critically important information [56]. These data would help prevent over-exploitation of aquifers or, where over-exploitation has already occurred, inform the development of management policies aimed at groundwater recovery [56].

Future research could focus on the establishment of maintenance schedules based on the value of the Borehole Dynamic Health Indicator (BDHI), further enhancing system longevity and performance. Additionally, future investigations could also explore how the proposed photovoltaic pumping tests could be integrated into a more extensive approach that addresses the maintenance and health assessment of all components within the PVWPS, including the photovoltaic array, motor-pump, and piping system.

## 6 Conclusion

In this article, was proposed and described a new method for supervising the borehole's health of a photovoltaic water pumping system (PVWPS): photovoltaic pumping tests. These tests offer a cost-effective way to assess the condition of the borehole throughout the PVWPS lifetime without interrupting its operation. In addition, indicators were developed to enable the stakeholders to easily interpret photovoltaic pumping tests results and monitor the borehole's health over time. Serving as a diagnostic tool, these tests help identify potential borehole issues before they affect the PVWPS's performance and durability. Specifically, they enable the early detection of signs of borehole vulnerability or overuse, thereby avoiding costly repairs or system failures. If necessary, the use of PVWPS can be adjusted in line with the evolving borehole condition. Ultimately, these tests offer the potential for a positive societal benefit to the PVWPS users, providing them with a more sustainable water access.

The protocol proposed in this study has been applied to a PVWPS, which provides water to 280 inhabitants in a rural village of Burkina Faso. Results indicate that photovoltaic pumping tests allow to accurately determine borehole parameters, achieving a model fit with an average R^2^ of 0.99. Additionally, they provide a reliable estimate of the borehole water depth, with an average normalized root mean square error (NRMSE) of 2.8 %. The cost of a photovoltaic pumping test was estimated at $43, making it more affordable than standard pumping tests (multiple-step drawdown test: $511; long-term test: $2050). Furthermore, over a 10-year period (the estimated motor-pump lifespan), the cumulative cost of conducting photovoltaic pumping tests every two years is less than 10 % of the expense of replacing the motor-pump prematurely. Over a 50-year period (the estimated borehole lifespan), the total cost of biannual photovoltaic pumping tests amounts to only 13 % of the cost of drilling a new borehole. These findings thus illustrate the interest of performing photovoltaic pumping tests, in particular in terms of improved characterization of the borehole evolution and potential cost savings. Although the protocol has been applied to a specific PVWPS in this article, it is generic and transferable to other installations. The proposed photovoltaic pumping test can be of interest to any stakeholder looking to improve the sustainability of PVWPS such as local authorities, governments, non-governmental organizations and companies.

## CRediT authorship contribution statement

**Ange Sahuquet:** Writing – original draft, Visualization, Validation, Software, Resources, Methodology, Investigation, Formal analysis, Data curation, Conceptualization. **Simon Meunier:** Writing – review & editing, Visualization, Validation, Supervision, Resources, Project administration, Methodology, Investigation, Formal analysis, Conceptualization. **Judith A. Cherni:** Writing – review & editing, Visualization, Validation, Supervision, Resources, Project administration, Methodology, Investigation, Formal analysis, Conceptualization. **Anne Charpentier:** Writing – review & editing, Visualization, Validation, Software, Methodology, Investigation, Formal analysis, Data curation, Conceptualization. **Thomas Vezin:** Writing – review & editing, Visualization, Validation, Software, Resources, Methodology, Investigation, Formal analysis, Data curation, Conceptualization. **Arouna Darga:** Writing – review & editing, Visualization, Validation, Supervision, Resources, Project administration, Methodology, Investigation, Formal analysis, Conceptualization. **Guillaume Zuffinetti:** Writing – review & editing, Visualization, Validation, Methodology, Investigation, Formal analysis, Conceptualization. **Peter K. Kitanidis:** Writing – review & editing, Visualization, Validation, Supervision, Project administration, Methodology, Investigation, Formal analysis, Conceptualization. **Loïc Quéval:** Writing – review & editing, Visualization, Validation, Supervision, Resources, Project administration, Methodology, Investigation, Formal analysis, Conceptualization.

## Data availability

Data will be made available on request.

## Declaration of competing interest

The authors declare that they have no known competing financial interests or personal relationships that could have appeared to influence the work reported in this paper.
